# Advancing cellulose utilization and engineering consolidated bioprocessing yeasts: current state and perspectives

**DOI:** 10.1007/s00253-025-13426-0

**Published:** 2025-02-13

**Authors:** Jordan Fortuin, Lazzlo J. Hoffmeester, Letitia S. Minnaar, Riaan den Haan

**Affiliations:** Department of Biotechnology, University of the Western Cape, Bellville, South Africa

**Keywords:** *Saccharomyces cerevisiae*, Consolidated bioprocessing, Cellulase, Heterologous enzyme production, Yeast strain improvement

## Abstract

**Abstract:**

Despite the lack of implementation of consolidated bioprocessing (CBP) at an industrial scale, this bioconversion strategy still holds significant potential as an economically viable solution for converting lignocellulosic biomass (LCB) into biofuels and green chemicals, provided an appropriate organism can be isolated or engineered. The use of *Saccharomyces cerevisiae* for this purpose requires, among other things, the development of a cellulase expression system within the yeast. Over the past three decades, numerous studies have reported the expression of cellulase-encoding genes, both individually and in combination, in *S. cerevisiae*. Various strategies have emerged to produce a core set of cellulases, with differing degrees of success. While one-step conversion of cellulosic substrates to ethanol has been reported, the resulting titers and productivities fall well below industrial requirements. In this review, we examine the strategies employed for cellulase expression in yeast, highlighting the successes in developing basic cellulolytic CBP-enabled yeasts. We also summarize recent advancements in rational strain design and engineering, exploring how these approaches can be further enhanced through modern synthetic biology tools to optimize CBP-enabled yeast strains for potential industrial applications.

**Key points:**

*• S. cerevisiae’s lack of cellulolytic ability warrants its engineering for industry.*

*• Advancements in the expression of core sets of cellulases have been reported.*

*• Rational engineering is needed to enhance cellulase secretion and strain robustness.*

*• Insights gained from omics strategies will direct the future development of CBP strains.*

**Graphical Abstract:**

**Figure:**
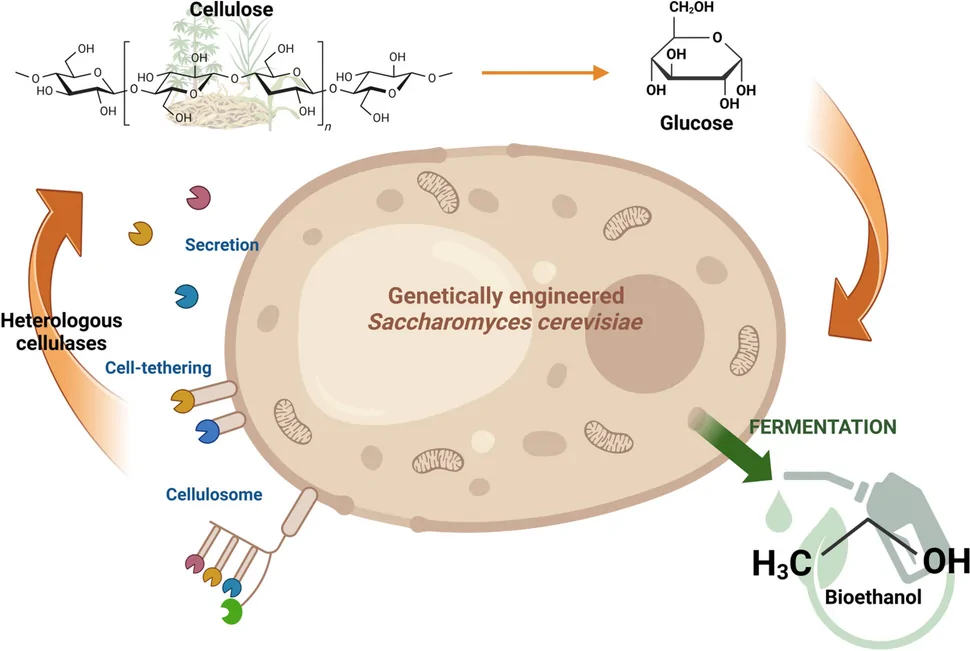

## Introduction

The growing need for energy security and economic stability, coupled with ongoing challenges in climate change, warrants intensified research into the future of global energy (Hassan et al. 2024). Accounting for 80% of all liquid biofuel production, bioethanol is currently the most sought-after and utilized alcoholic biofuel on the market (Jansen et al. 2017; Khan et al. 2021). This recent resurgence in demand for bioethanol can be attributed to the steadily decreasing fossil fuel reserves, increasing populations, rapid urbanization, the volatile price of oil, and the need for clean energy (Melendez et al. 2022). First-generation (1G) bioethanol is produced from edible crops, and while processing from feedstock to product is relatively simple and well-established, the use of edible feedstocks has led to a “food vs fuel” debate, due to upscaled 1G bioethanol production pressurizing the already-pressured food supply, with additional impacts on land and water usage (Lugani et al. 2020; Nigam and Singh 2011; Niphadkar et al. 2018). Moreover, the price of 1G feedstocks can fluctuate, potentially impacting the profitability of the process further (Favaro et al. 2019).

The drawbacks of 1G bioethanol have led to increased interest in second-generation (2G) biofuel production. 2G bioethanol makes use of non-edible, low-cost, and renewable lignocellulosic biomass (LCB) feedstocks, eliminating the competition between energy and food production (Niphadkar et al. 2018; Olguin-Maciel et al. 2020). The biochemical composition of LCB includes the energy-rich biopolymers lignin, hemicellulose, and cellulose, the microfibrils of which are tightly braided together, providing structure and rigidity to the cell wall of plants (Mujtaba et al. 2023). The associated drawbacks of 2G biofuel production include the recalcitrance of LCB feedstocks which necessitates costly pre-treatment methodologies to facilitate the separation of lignin from the carbohydrate-rich cellulose and hemicellulose fractions, allowing them to be hydrolyzed to fermentable sugars (Lugani et al. 2020; Van Dyk and Pletschke 2012). In addition, 2G bioethanol production requires enzyme cocktails with effective proportional ratios of cellulases, which act synergistically to completely saccharify lignocellulose polysaccharides to simple sugars which can be fermented to ethanol by a suitable microorganism(s) (Dadwal et al. 2021). However, this practice constitutes a major obstacle for 2G bioethanol production due to the exorbitant costs of commercial cellulases and large load requirements for complete feedstock conversion (Sharma et al. 2020).

**Fig. 1:**
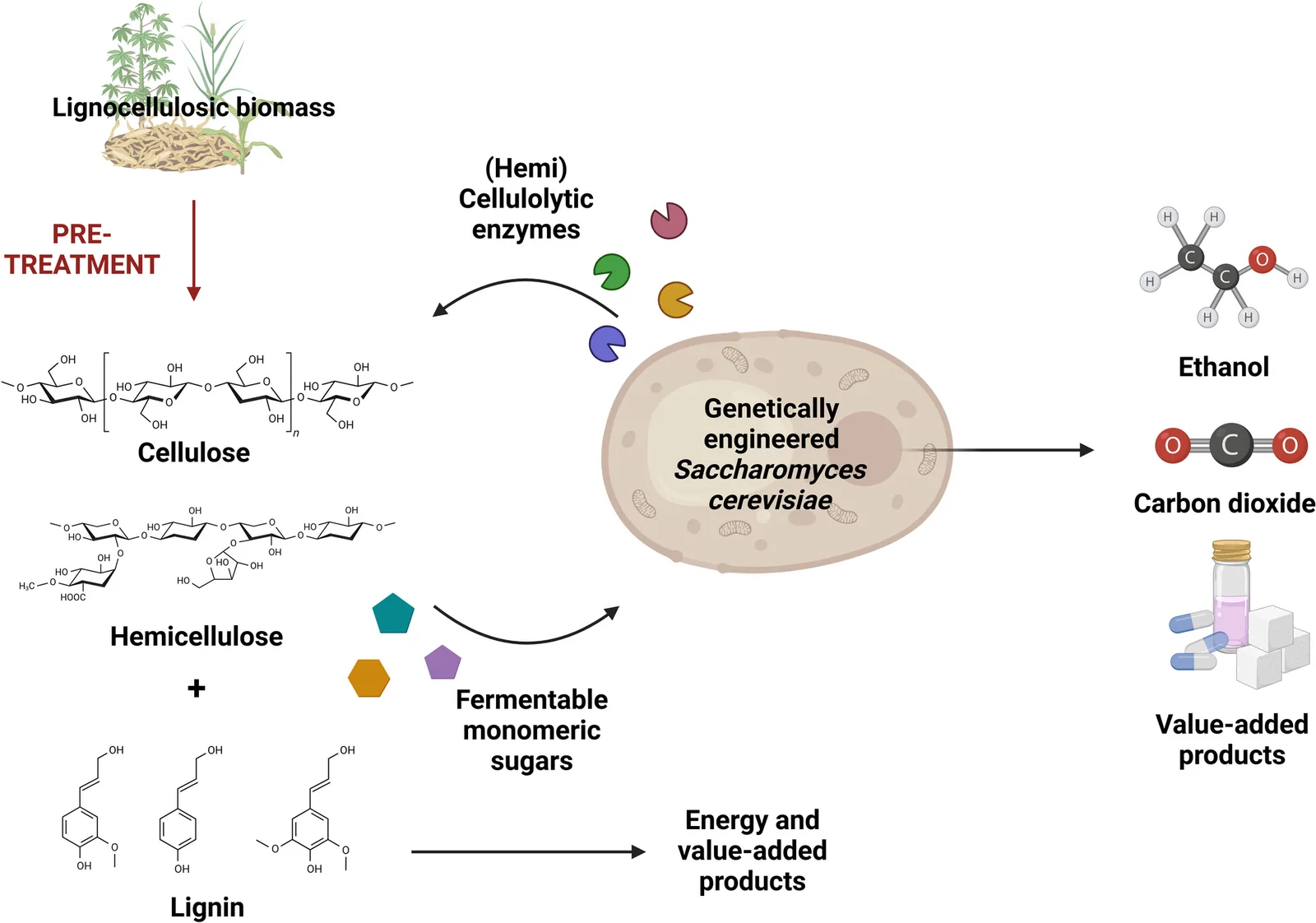
Consolidated bioprocessing (CBP) involves the direct microbial conversion of lignocellulosic biomass (LCB) into ethanol and other value-added products. While *Saccharomyces cerevisiae* possesses several advantageous properties for this process, it requires substantial genetic engineering to enhance its suitability, particularly by enabling cellulolytic activity. Created in BioRender.com

To enhance biomass conversion, combining polysaccharide hydrolysis with hexose and pentose fermentation within a single organism is envisioned (Fig. 1). This approach streamlines the conversion of biomass to bioethanol through a process known as consolidated bioprocessing (CBP)—often referred to as the “holy grail” of low-cost biomanufacturing. By integrating these steps, CBP eliminates the need for exogenous enzymes, significantly reducing production costs (Claes et al. 2020; Davison et al. 2019a; Minnaar et al. 2024). The yeast *Saccharomyces cerevisiae* has long since been used in the production of fermented foods and beverages and has also emerged as a major microorganism used in industrial enzyme and biofuel production (Den Haan et al. 2021). With its numerous advantageous characteristics as an industrial ethanol producer—including high productivity, robustness, a fully mapped genome, and amenability to genetic engineering—*S. cerevisiae* is a promising candidate for genetic engineering and further development for industrial CBP (Den Haan et al. 2021; Zhao et al. 2024).

This review will focus on the establishment of cellulose utilization in yeasts for industrial CBP implementation. We discuss the engineering of different cellulase systems into yeast and reports of their application as CBP organisms. We also examine strategies to further enhance CBP via improvement of gene transcription, enzyme production, and secretion, as well as strategies to improve yeast robustness to industrial CBP stresses.

## Cellulase systems and strategies to engineer them into yeast

**Fig. 2:**
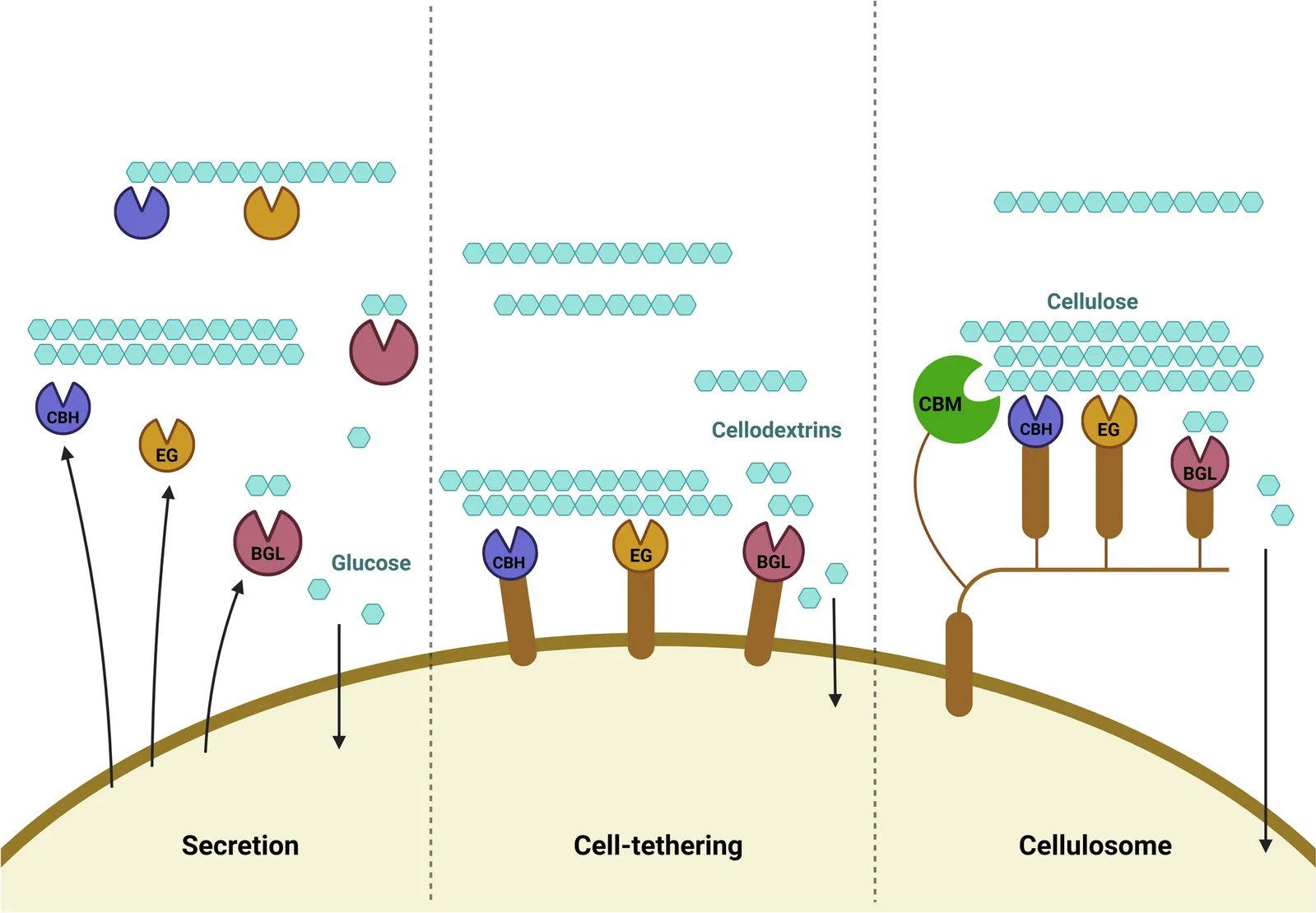
The three strategies generally reported to enable the production of a basic cellulolytic system in yeasts include the secretion of free enzymes, cell tethering of individual enzymes, and the attachment of cellulases to cell-tethered scaffold proteins to form a cellulosome. Combinations of the secretion and cell tethering strategies have also been reported. CBH, cellobiohydrolase; EG, endoglucanase; BGL, β-glucosidase; CBM, carbohydrate-binding module. Created in BioRender.com

Cellulose is the most abundant biopolymer in LCB, with a well-defined structure comprised of β−1,4-linked glucose monomers, constituting the major fraction of fermentable sugars in LCB (Van Zyl et al. 2015; Zhou et al. 2017). These glucose chains are linked by strong hydrogen bonds to form microfibrils of cellulose chains, making the cellulose crystalline and highly resistant to degradation (Yousuf et al. 2020). Assimilation of cellulose requires an organism to express enzymes to break down cellulose components (cellodextrins) into monomeric glucose to enter cellular metabolism through glycolysis (Lane et al. 2018). To conduct the successful saccharification of insoluble cellulose, *S. cerevisiae* must thus be engineered to express at least a core set of cellulase-encoding genes—cellobiohydrolases (CBHs), endoglucanases (EGs), and β-glucosidases (BGLs)—and subsequently produce enzymes at sufficient titers and optimal ratios (Claes et al. 2020; Wightman et al. 2020). These cellulases can be engineered to be (i) secreted freely, (ii) directly cell wall-tethered, or (iii) indirectly surface attached via a cellulosome (Valenzuela-Ortega and French 2019) (Fig. 2).

The cellulosome is a multi-enzyme complex consisting of a cell-wall-anchored scaffoldin protein which houses multiple cellulases bound via various dockerins and cohesins, and a carbohydrate-binding molecule (CBM) which binds the complex to the cellulosic substrate (Oh and Jin 2020). So far, only “mini-cellulosome” versions (displaying few cellulases) of these cellulosomes have been heterologously expressed in yeast. However, Anandharaj and co-workers (2020) recently developed a *Kluyveromyces marxianus* strain to display the so far largest known cellulolytic complex and demonstrated the production of 3.09 and 8.16 g/L ethanol from Avicel and phosphoric acid swollen cellulose (PASC), respectively, the highest ethanol titers of any known cellulosome producing yeast.

Secretion is often the easiest of the three methods to implement (Lane et al. 2018). With no restrictions on enzyme localization outside the cell, simpler expression cassette construction, and the primary limitation being the enzyme production capacity, secretion frequently results in high levels of enzyme production and efficient diffusion. Secreted enzymes are also reported to penetrate the secondary cell wall of plant biomass, increasing the yeast cell’s accessibility to cellulose, which is important for hydrolysis (Liu et al. 2015; Oh and Jin 2020). However, free-secreted cellulases often lose functionality and diffuse away over time. Thus, they present no direct advantage to the cells that produced them and reduce the practicality of the industrial process as these enzymes are not recyclable. Contrastingly, cell-tethering immobilizes cellulases to the cell wall and is often reported to present increased thermostability and cellulase activities despite enzyme carrying capacity being limited to the available cell surface area (Den Haan et al. 2015; Ueda 2016). Tethering enzymes to the cell also allows the re-use of the cells and their attached enzymes in multi-batch fermentations, reducing the cost of both yeast propagation and supplementation with exogenous enzymes (Liu et al. 2015). In addition, having the required cellulases tethered in proximity may allow for synergistic cellulose degradation as well as for easier glucose uptake when glucose monomers are released in the vicinity of the cell (Oh and Jin 2020; Van Zyl et al. 2015). However, this also means that tethered enzymes will only be able to interact with substrate in the vicinity of the cell. It was reported that cell-surface-displayed enzymes impose a greater metabolic burden on the cell compared to secreted enzymes (Ding et al. 2018). This burden increased with gene copy number and was independent of plasmid-based or chromosomal integration methodologies. The glycosylphosphatidylinositol (GPI) anchoring system is one of the most used cell tethers but is a complex and metabolically expensive system. With both secretion and cell-anchoring having their own advantages and disadvantages, their combined use may be more efficient than either used on its own (Liu et al. 2015).

## Engineering CBP-enabled yeasts

With the advent of synthetic biology, various research efforts have shown the immense potential of *S. cerevisiae* to be genetically modified for use in cellulosic ethanol production processes. By expressing a core set of heterologous cellulases using the strategies mentioned above, significant progress has been made towards establishing cellulose utilization in yeasts (Table 1). Despite the difficulty in comparing these results due to variation in substrates used, they show that cellulosic ethanol production is possible using recombinant yeasts capable of synthesizing a core complex of (hemi)-cellulolytic enzymes via different expression strategies. However, several challenges impede the sustainable implementation of this process on an industrial level. One of the main challenges involves the low heterologous protein titers and subsequent ethanol yields obtained by the recombinant hosts (Davison et al. 2019a). To overcome this problem, integrating cellulase-encoding genes in optimal ratios in combination with different expression strategies has been attempted, with some significant successes. Recently, multiple (hemi)-cellulase-encoding genes were integrated into the genome of an industrial strain, through multiple rounds of CRISPR-Cas9-based transformations (Claes et al. 2020). A total secreted cellulase activity of 94.5 FPU/g DCW was achieved, which enabled the direct conversion of LCB into ethanol, without the addition of exogenous cellulase cocktails. Using this strain, complete hydrolysis of cellobiose and corncob xylan was achieved with an ethanol productivity of 4.46 g/L/h, while partial conversion of both solid and liquid fractions of hydrothermally pre-treated sugarcane bagasse was achieved in just 30 h, with an ethanol productivity of 1.86 g/L/h (Perez et al. 2023).

**Table 1:** Establishment of cellulose utilization in yeast through the production of a core set of cellulases. Refer to the studies mentioned for details on the origins of the various genes expressed. Studies reporting the conversion of cellulosic substances without the addition of exogenous enzymes are shown

Strategy/application	Ethanol titer/yield	% of the theoretical ethanol maximum	Reference
*S. cerevisiae*			
Cell-attached expression of EG, CBH, and BGL via delta-integration	2.9 g/L from PASC within 40 h	PASC 88.5%	Fujita et al. 2004
Cell-attached expression of EG, CBH1, CBH2, and BGL	6.5 g/L from PASC 1.4 g/L from Avicel 1.3 g/L from liquid hot water (LHW) pre-treated rice straw (after 96 h)	PASC 66% Avicel 27% Rice straw 7%	Liu et al. 2016
Cocktail delta-integration to display EG, CBH1, CBH2, and BGL	2.9 g/L from Avicel 0.8 g/L from pre-treated rice straw (after 96 h)	Avicel 57%	Liu et al. 2017
Display EG, CBH, and BGL, individually on the cell surface, in optimal ratios (consortia)	1.8 g/L from PASC after 84 h	PASC 82.3%	Mo et al. 2015
Co-culture of (hemi)-cellulolytic Y5 strains	2.11 g/L from PASC 1.6 g/L from steam-exploded corn stover (after 96 h)	PASC 80.2% Corn stover 64.7%	Chen et al. 2018
Secretion of EG and BGL via natural isolate YI13	4.05 g/L ethanol from pre-treated corn cob after 168 h	Corn cob 83.7%	Davison et al. 2019b
Enzyme density optimized surface display of mini-cellulosomes with CBH1, CBH2, EG, and BGL	4.92 g/L from PASC after 168 h	PASC 96%	Ma et al. 2024
Secretion of fungal-derived CBH1, CBH2, and EG with cell-tethered BGL in natural *S. cerevisiae* isolates	4–4.5 g/L from Avicel after 120 h	Avicel 40%	Minnaar and Den Haan 2023
*K. marxianus*			
Cellulosome display of BGL, CBH, EG, LPMO and CDH	8.61 g/L from PASC after 120 h 3.09 g/L from Avicel after 144 h	N/A	Anandharaj et al. 2020

While free (secreted) enzymes experience no limitation with regard to the physical surface area of the host cells, synergy among enzymes is hampered by enzyme detachment from cellulosic substrates (Oh and Jin 2020). Surface-immobilized cellulases are in closer proximity which alleviates synergism constraints. This was illustrated in a study conducted by Ma and co-workers (2024), where a mini-cellulosome was displayed on the yeast cell surface at maximum enzyme density capacity. The authors identified molecular crowding as the main cause for suboptimal enzyme density displayed on the cell surface at a single-cell level, which was improved by increasing the integrated gene copy number. Subsequent fermentation on 1% PASC showed a cellulose-to-ethanol yield of 4.92 g/L (96% of the theoretical maximum) at 144 h. The potential of natural *S. cerevisiae* isolates to secrete a core set of cellulases was recently assessed (Minnaar and Den Haan 2023). While high levels of heterologous protein production were linked to a reduced maximum growth rate and minor changes in overall strain robustness, superior secretion capacity for the heterologous cellulases was observed compared to domesticated strains. When cultivated on crystalline cellulose (Avicel) under oxygen-limited conditions, the superior cellulase secretors YI13_BECC and YI59_BECC achieved ethanol concentrations between 4 and 4.5 g/L after 120 h, representing 35–40% of the theoretical maximum ethanol yield, without the need for exogenous enzymes. While these studies signify the potential use of yeast as a cell factory for cellulosic ethanol production via CBP, incomplete saccharification and low ethanol yields from both purified (laboratory-grade) and mixed (LCB) cellulose streams remain a great hurdle.

### Conversion of LCB substrates with CBP-enabled yeast and decreased cellulase loads

To render ethanol production on an industrial level economically feasible, ethanol concentrations and yields of > 40 g/L and > 70% of the theoretical maximum are required (Van Dyk et al. 2023). To achieve this, current production processes use exogenous cellulase cocktails to aid in improving the hydrolysis of LCB (Table 2). In one such study, Van Dyk and co-workers (2023) showed that ethanol yields in excess of 10 g ethanol/100 g dry paper sludge (PS) could be achieved when high-solid loadings of PS waste were hydrolyzed by an industrial strain, namely Ethanol Red®, with the addition of 10 FPU/g dry PS. Strikingly, submerged fermentations with Cellusec 1.0® and Cellusec 2.0® (Ethanol Red® strains engineered to contain hemi- and/or -cellulolytic enzyme activities) yielded improved ethanol yields (> 20 g ethanol/100 g dry PS) with a 50% reduction in exogenous cellulase cocktail additions. Li and co-workers (2023a) used a mutant *S. cerevisiae* LF1 strain capable of xylose utilization to ferment pre-treated and detoxified corn fiber hydrolysate (20% solid loading, 20 FPU/g solids). An ethanol concentration of 37.2 g/L was obtained, which corresponded to 92% of the theoretical maximum value. Additionally, the ability of a *S. cerevisiae* strain, engineered to co-utilize glucose and xylose, was tested for converting high solid loadings of hydrothermally pre-treated sorghum into ethanol (Cheng et al. 2021). After 96 h, a maximum ethanol titer of 50.1 g/L was achieved, with an ethanol productivity of 0.8 g/L/h. This corresponded to 73.3% of the theoretical maximum, although a high loading of exogenous cellulase was required. Adaptive laboratory evolution (ALE) was shown to be an effective method for increasing the thermotolerance of a cellulolytic *S. cerevisiae* strain (Zhang et al. 2023a). This approach enabled a 127% increase in ethanol production from pre-treated wheat straw maintained at 50 °C, achieved within just 24 h.

**Table 2:** Yeast strain constructs utilized in second generation bioethanol production with the incorporation of exogenous enzymes

Strain	Features/description	Reference
Cellusec 1.0®	*S. cerevisiae* strain engineered for xylose consumption (NovelYeast, Leuven-Heverlee, Belgium)	Van Dyk et al. (2023)
Cellusec 2.0®	*S. cerevisiae* strain engineered for xylose consumption and secretion of the cellulases CBH1 and EG (NovelYeast, Leuven-Heverlee, Belgium)	Van Dyk et al. (2023)
*S. cerevisiae* LF1	Industrial strain engineered for efficient co-utilization of glucose and xylose	Li et al. (2023a)
*S. cerevisiae* SR8 Δ ADH6	Engineered for co-fermentation of glucose and xylose (CABBI, Urbana, IL, USA)	Cheng et al. (2021)
*S. cerevisiae* Z100	Highly thermotolerant xylose utilizing strain obtained through engineering, and ALE on wheat straw hydrolysates at high temperatures	Zhang et al. (2023a)

While these studies highlight the significant impact of exogenous cellulase cocktails in improving the ethanol yields and titers during fermentations, the high costs implicated by its utilization still render cellulosic ethanol production on an industrial level economically unfeasible. Therefore, Liu and co-workers (2023) investigated how selective hydrolysis of cellulose can be achieved with the use of a catalytic accelerator, instead of exogenous cellulase cocktails. Using this approach, co-fermentation on the resulting corn fiber hydrolysates delivered an ethanol yield of 92% of the theoretical maximum. This proved to be an effective and potentially economically beneficial way to improve substrate saccharification for cellulosic ethanol production.

Although great strides have been made in improving cellulose conversion, strain performance during fermentation is greatly affected by factors like inhibitors introduced during pre-treatment and the harsh overall fermentation conditions (Brandt et al. 2021a). To address these challenges, the immobilization of yeast cells could preserve cellular metabolism and vitality during fermentations. Malik and co-workers demonstrated that immobilization of a co-culture of yeast cells (*S. cerevisiae* and *Pachysolen tannophilus*) in alginate beads provided a protective barrier which allowed cells to perform optimally for up to 5 cycles of fermentation on pre-treated cotton stalk (Malik et al. 2021). Regardless of the type of pre-treatment used, an ethanol productivity of 0.44–0.46 g/g was achieved, with a greater than 90% sugar utilization efficiency, with the addition of exogenous cellulase cocktails (25 FPU/g solid substrate). Ramos and co-workers (2023) tested the application of immobilized CBP-enabled strains on cellulosic substrates. They showed that a mixture of glucose, xylose, cellobiose, xylan, and carboxymethylcellulose could be efficiently converted to ethanol, though some diffusion effects were noted. However, the conversion of crystalline cellulose substrate was not reported.

## Rational engineering of gene cassettes for the improvement of CBP yeasts

Despite the successes achieved in CBP yeast construction, substrate conversion remains limited by the inherently low protein secretion capacity of yeast (Kroukamp et al. 2018). This results in suboptimal cellulase production, highlighting the need for further genetic engineering to enable efficient industrial implementation. Promoter systems are among the most critical elements requiring optimization, particularly to achieve high-level production and balanced expression of heterologous cellulases in CBP-enabled yeast. This will help ensure a high enzyme titer with cellulase components in optimal ratios.

### Improved promoter systems

Constitutive promoters are often preferable to inducible promoters in most metabolic engineering applications as they are reported to maintain relatively stable levels of expression in contrast to their inducible counterparts (He et al. 2023; Hubmann et al. 2014; Tang et al. 2020). When implementing promoters for heterologous gene expression, the promoter choice is critical (Den Haan et al. 2021; Xiong et al. 2018). While the strength of the promoter directly affects the intensity of gene expression, even constitutive promoters—despite their designation—are condition-specific, and their expression levels may fluctuate under different environmental conditions (He et al. 2023; Nambu-Nishida et al. 2018; Xiong et al. 2018). Despite the numerous reports on promoter performance, variation in experimental setup impacts the comparability across studies (Hubmann et al. 2014). Considering the sensitivity of promoters, determining the overall “best” promoter has remained elusive, and therefore, it may be more fitting to assess endogenous promoters under the exact conditions for which they are intended (Den Haan et al. 2021; Myburgh et al. 2023). In the context of 2G CBP fermentation, this would typically involve anaerobic or micro-aerobic conditions with low levels of free monomeric sugars, elevated temperatures, and the presence of stress-inducing metabolites. Promoter performance is typically evaluated by researchers using reporter genes, such as yeast-enhanced green fluorescent protein (yEGFP) or *lacZ*, to compare promoter activity in laboratory yeast strains under various cultivation conditions (Myburgh et al. 2020; Nambu-Nishida et al. 2018; Peng et al. 2015; Xiong et al. 2018). However, in a notable example where promoters were assessed under CBP-associated conditions, Xiong and co-workers investigated the activity of eight endogenous promoters and one hybrid promoter in the presence of xylose and under stress conditions associated with the fermentation of lignocellulosic hydrolysates (3.6 g/L acetic acid, 1.0 g/L furfural, and temperatures of 39 °C). The constitutive *TDH3* promoter and the hybrid *3XC-TEF1* promoter (containing a *TEF1* promoter core and three tandem UASs of the *CLB2* promoter) resulted in the best activity under the chosen conditions.

In attempts to overcome the shortcomings of natural promoters, already-strong promoters are being modified to further improve their performance, creating synthetic promoters. Methods of creating synthetic promoters include, but are not limited to, random mutation of promoter sequences, rational promoter design via computational methods, the addition of introns to promoter sequences, and fusing promoter elements to create hybrid promoters (reviewed in depth by He et al. 2023) (Table 3). Although random mutagenesis can generate genetic diversity, its results can be unpredictable, and its screening processes are labor-intensive (Nguyen et al. 2024). With promoters becoming better characterized over time, synthetic promoters are more commonly made through systematic and rational design via computational modelling and simulation (Hubmann et al. 2014; Myburgh et al. 2023). This method requires prior knowledge of the structure and function of promoter elements, but these are becoming more accessible over time, allowing for specific tailoring and more precise control of promoter function (Feng and Marchisio 2021b; Nguyen et al. 2024). Kotopka and Smolke (2020) attempted to determine the sequence-function relationship between conserved promoter motifs and the spacer sequences between them. Fluorescence-activated cell sorting and next-generation sequencing were performed on two promoter libraries (675, 000 constitutive and 327, 000 inducible) and the resulting data was used to develop a convolutional neural network (CNN) model trained to accurately predict promoter activity (*R*^2^ > 0.79). This work provided promoter sets with valuable properties for synthetic biology applications, along with a tool for generating promoters with desirable characteristics.

**Table 3:** Summary of synthetic promoter techniques applied to improve heterologous protein production

Technique	Concept	Applications	Reference
Random mutagenesis	Endogenous promoter is subjected to random mutations	Random mutation of the core promoter region of *PFY1*_P_ to create a diversified promoter library	Blount et al. 2012
Rational engineering/total de novo synthesis	In silico design of completely synthetic promoters	Construction of minimal constitutive and inducible yeast promoters (< 120 bp) as strong as *TDH3*_P_ and *GAL1*_P_, respectively Utilization of a model-guided approach to predict promoter activity based on predicted nucleosome affinity to be used in the redesign of endogenous promoters and de novo design of synthetic promoters—resulted in the construction of six fully synthetic promoters	Redden and Alper 2015 Curran et al. 2014
Intron addition	Introns, which function as gene regulators, are fused to promoter sequences	Addition of the *RPS25A* intron improved 5 out of 6 promoters expressing α-amylase Creation of a library of 72 intron-aided promoters, improving the dynamic range of the promoters from 2.4- to sevenfold. This was then used to train a predictive model of intron-promoter binding to improve heterologous gene expression	Myburgh et al. 2020 Cui et al. 2021
Hybrid promoters	Merging of promoter core elements with enhancer elements of different sources	Addition of upstream activating sequence (UAS) upstream of core sequences of “strong” promoters further enhanced promoter activity, expanding the transcriptional capacity of *TDH3*_P_ by 2.5-fold Extension of a viral core promoter with short sequences of the *CYC1* yeast promoter, creating hybrid promoters—the strongest of which outcompeted *TEF2*_P_	Blazeck et al. 2012 Feng and Marchisio 2021a

The correlation between introns and the regulation of gene expression has presented a potential means by which introns can further improve the strength of promoters (He et al. 2023). A study by Myburgh and co-workers (2020) involved the addition of the *RPS25A* intron to endogenous promoters which improved the expression of heterologous *Aspergillus terrus* α-amylase in most cases. Following this, strains were constructed to co-express chosen α-amylase cassettes and a gluco-amylase to be used in the fermentation of raw starch and broken rice, reaching a 97% of the theoretical ethanol yield and converting 100% of the available carbon products in 120 h. The construction of hybrid promoters presents a promising technique for enhancing gene expression by providing a broader range of strength and control (Myburgh et al. 2023). Additionally, the application of synthetic promoter design techniques to these hybrid promoters could adapt them to specific requirements, potentially eliminating the drawbacks associated with hybrid promoters such as unwanted transcription factor binding sites or other elements. This way, promoters could contain the benefits of both hybridized biological promoters and synthetic promoter elements (Nguyen et al. 2024). However, it is important to emphazise that expression levels should be tested and optimized under conditions relevant to the desired process. As each promoter engineering strategy has its own focuses, a combinatorial approach may provide the constructed promoter with the benefits of the combined strategies, possibly increasing the potential of promoters to help overcome the limitations faced by current CBP-enabled yeast strains (He et al. 2023).

### Optimized signal peptides for improved cellulase secretion

*S. cerevisiae* secretory expression of heterologous proteins is subject to bottlenecks (e.g., plasmid vector systems, cultivation conditions, and properties of the target protein(s)), limiting their yield (Cho et al. 2022). Signal peptides, located on the N-terminus of nascent polypeptides, act as recognizable elements for transportation to the cell wall or endoplasmic reticulum through secretory pathways. More simply, they determine the secretion pathway of proteins and direct them to their required destination, hence affecting secretion efficiency. Once the protein has been properly directed, the signal peptide is removed by signal peptidase to allow the protein to be properly folded and moved to its destination (Aza et al. 2021; Inokuma et al. 2016; Xue et al. 2023). The use of signal peptides, both natural and synthetic, for the enhancement of heterologous gene expression has been successful in many cases. Replacement of the native signal peptide of an *egI* with the native *S. cerevisiae* MFα1 pre-pro-leader sequence resulted in a yeast strain showing 61.5% higher EG activity (Zhu et al. 2010). Aza and co-workers (2021) recently optimized the sequence of the MFα1 pre-pro-leader through iterations of directed evolution, ultimately creating a final optimized α-pre-pro-leader sequence which resulted in notable increases in the secretion of both fungal oxidoreductases and hydrolases in *S. cerevisiae.* Interestingly, Inokuma and co-workers (2016) demonstrated that the use of the SED1-derived signal peptide resulted in enhanced heterologous BGL and EG activities in *S. cerevisiae*, outperforming the commonly used GLUA and MFα1 leader peptides.

Xue and co-workers (2023) conducted a genome-wide systematic bioinformatics analysis and evaluated the secretion capacity of signal peptides from *S. cerevisiae*. Their study highlighted key features of signal peptides, including region properties, consensus motifs, evolutionary relationships, and codon bias. Additionally, they demonstrated that using different signal peptides for heterologous protein secretion can trigger diverse cellular metabolic responses. These findings suggest that further systematic testing of signal peptide candidates could enhance heterologous cellulase production in yeast. However, like promoters, the secretion efficiency of signal peptides has been shown to be dependent on its target protein (Inokuma et al. 2016), so a “universal” signal peptide for the improvement of yeast secretion does not yet exist (Aza et al. 2021).

## Rational engineering of yeast strains for improvement of CBP yeasts

Protein production utilizes various cellular machinery components to perform metabolic functions and is intricately regulated at multiple stages, starting from the earliest points of transcription and translation (Zhao et al. 2024). Rational strain engineering strategies have enabled the optimization of the protein secretion pathway of recombinant *S. cerevisiae* strains engineered to produce heterologous cellulases.

### Rational engineering of the protein secretion pathway

In the yeast secretory pathway, proteins—native or heterologous—must traverse the intracellular components of the cell before they can be released. After transcription and translation, newly synthesized polypeptides are transported from the endoplasmic reticulum (ER) to the *cis* Golgi; they are then transported through the compartments of the Golgi and eventually sorted by the *trans*-Golgi network into secretion vesicles which are bound for certain organelles or the cell membrane (Kroukamp et al. 2018). The yeast secretory pathway offers numerous genetic engineering targets for enhancing secretion efficiency, which have been successfully exploited in various studies to improve heterologous protein secretion (Table 4). Li and co-workers (2022) recently developed a genome-scale model for protein secretion in *S. cerevisiae* (pcSecYeast). This computational tool can be used to simulate endogenous and heterologous protein secretion as well as its interaction with the yeast metabolism and gene expression. pcSecYeast is useful in the rational design of recombinant protein production in *S. cerevisiae*, especially considering the challenges associated with the complexity of the yeast secretory pathway, to both guide yeast engineering and enhance protein production.

**Table 4:** Gene targets along the protein secretion pathway for enhanced heterologous hydrolase secretion

Protein secretion pathway segment	Gene target	Function	Observed effect	Reference
Transcription and translation	Protein secretion enhancer 1 (*PSE1*) over-expression	Facilitates export of mRNA from the nucleus to the cytosol, increasing expression of multiple secretion pathway genes	Increased BGL, EG, and CBH activities by 3.7-, 1.25-, and 1.2-fold respectively in *S. cerevisiae* Y294	Kroukamp et al. 2013
Translocation through the ER	*SRP14* and *SRP54* over-expression	Co-translational translocation components	Improved heterologous BGL (44% and 56%, respectively) and EG (18% and 16%, respectively)	Tang et al. 2015
ER folding	*PDI1* and *ERO1* over-expression *PDI1* over-expression	Encodes a protein disulfide isomerase, and sulfhydryl oxidase	Resulted in a 7.8-fold increase in heterologous amylase-human serum albumin and an 18.2-fold increase in albiglutide	Li et al. 2023b
			Increased activity levels of BGL CBH and EG	Song et al. 2018
Unfolded protein response (UPR) pathway	Factors relating to *HAC1* gene-combined modifications	Transcription factor in the UPR	Produced 3.3-fold more α-amylase than the control strain	Lin et al. 2023
Golgi processing and transport	*PMR1* disruption	Encodes a Ca^2^⁺/Mn^2^⁺ ATPase of the Golgi membrane; a key factor in ER folding, protein sorting, and glycosylation	Alleviated over-glycosylation; resultant strains displayed increases in individual BGL, CBH, and EG activities and improved degradation of multiple cellulosic substrates	Song et al. 2018
	*OCH1* and *MNN9* disruption	Encodes α−1,6-mannosyl-transferases of cis-Golgi and Golgi complex	*OCH1* 170%, 140%, and 160% increase in BGL, EG, and CBH activities, respectively *MNN9* 160%, 176%, and 102% increase in BGL, EG, and CBH activities, respectively	Tang et al. 2016
	SNARE complex proteins: *SED5* and *SSO1* over-expression *SED5* over-expression	Facilitates transport of quality-controlled heterologous proteins to the Golgi	Enhanced heterologous BGL activity ranging between 22 and 130% *SED5* over-expression also led to significant CBH secretion enhancement	Van Zyl et al. 2016
			Enhanced crystalline cellulose hydrolysis of a ratio-optimized cellulase consortium displaying strain	Chetty et al. 2022
Vesicle trafficking	*SLY1* and *SEC1* (of the SEC1/MUN18 complex) over-expression	Functions towards tethering and fusion of vesicles	Improved secretion of heterologous α-amylase by 43% and 62%, respectively	Hou et al. 2012
	*SEC4* (Rab-related protein) over-expression	Facilitates intracellular protein trafficking throughout the protein secretion pathway	Demonstrated a threefold increase in α-amylase activity	Toikkanen et al. 2003
			16% and 22% increase in BGL and EG activity	Tang et al. 2017
Vacuole protein trafficking	*VPS5* and *VPS17* disruption	Encodes a protein receptor responsible for the seizing and escorting of carboxypeptidase Y and abnormal proteins to the vacuole	~ 80% increase in α-amylase activity	Huang et al. 2018

### Rational engineering of cell wall-related genes to improve cell-tethered cellulase display capacity

A significant limitation of utilizing cell-tethered heterologous protein systems to enable cellulase activity in yeast is the finite surface area of the cell wall, which restricts enzyme display capacity. This, in turn, hampers hydrolytic activity and reduces overall product formation (Yamada et al. 2013). While research in this area is still limited with much to be elucidated, studies in the systematic genetic modification of cell wall environments and biosynthesis have shown promise in enhancing cell-tethered cellulase activities. The disruption of the *MNN2* gene (encoding an α−1,2-mannosyltransferase) led to increased cell-tethered EG and BGL activities attributed to this deletion increasing β−1,6-glucan levels, availing more linking sites for GPI anchors of heterologous fusion proteins (Matsuoka et al. 2014). Similarly, the major non-enzymatic GPI cell wall protein (GPI-CWP) was disrupted by knocking out the *CCW12* and *CCW14* genes, resulting in increased cell wall thickness, which enhanced the yeast’s capacity for heterologous cellulase display, confirmed by a 1.4-fold increase in BGL activity in the GPI-CWP co-knockout strain (Inokuma et al. 2021). The disruption of *SED1* (which encodes a stress-induced structural GPI-CWP) was shown to increase cell-surface attached BGL activity by 22%, highlighting the potential benefit of reducing anchorage competition within the limited yeast cell wall capacity (Bamba et al. 2018). Furthermore, it was demonstrated that the over-expression of the GPI biosynthesis-related gene *LAS21*, resulted in a 1.5-fold increase of cell surface displayed BGL activity (Arnthong et al. 2022). Recently, Chen and co-workers (2023) showed that the inactivation of *DFG5*, *YPK1*, *FYV5*, *CCW12*, and *KRE1* improved BGL secretion and surface display. Further improvements were shown with *ΔFYV5 ΔCCW12* double disruption, which also improved CBH secretion.

### Rational engineering and evolutionary methods to improve stress tolerance

The fermentation environment in 2G biofuel production is inherently stressful for the fermentative organism due to factors such as elevated temperatures, organic acids, nutrient limitations, and osmotic stress (Brandt et al. 2021a). Moreover, chemical or physico-chemical pre-treatment-derived inhibitors cause additional stress to the yeast. Inhibitors can include phenolic compounds, furan aldehydes, carboxylic acids, and soluble sugars derived from lignin degradation, pentose, and hexose degradation, by-products of hemicellulose and furan derivatives, and intermediate and end products of hydrolyzed lignocellulosic materials, respectively (Kim 2018; Baruah et al. 2018). The establishment of a cellulase system in yeast is therefore not sufficient to create an industrial CBP organism. Various methods are frequently employed to enhance tolerance, including the over-expression of structural genes related to resistance, long-term adaptive evolution, artificial mutagenesis, and genome shuffling (Saini et al. 2018). ALE techniques are frequently used to enhance the tolerance of yeast strains where the yeast acquires advantageous traits over several generations of cultivations under premeditated selective pressures (e.g., heat, pH, and toxic compounds) (Mohedano et al. 2022). ALE was used in the serial cultivation of *S. cerevisiae* CEN.PK113-7D subjected to increasing concentrations of dicarboxylic acids to improve tolerance (Pereira et al. 2019). Reverse engineering determined that the mutation in the evolved strain was likely due to the upregulation of multidrug transporters encoded by *PDR12* and *QDR3.* ALE necessitates meticulous design due to potential trade-off effects that may result in the loss of crucial industrial properties. Additionally, understanding the conferred adaptations of evolved strains requires intense multi-omics screening to elucidate acquired mutations, which is often time-consuming and costly (Mohedano et al. 2022; Xu et al. 2024). However, this technique has revealed multiple gene targets which confer enhanced strain robustness, tolerance, and resistance to environmental stress factors (Brandt et al. 2021a; Oh and Jin 2020). For example, *ESBP6* was identified as a gene target in improving aromatic acid tolerance and robustness in product formation (Pereira et al. 2020), and *ASG1*, *ADH3*, *SKS1*, and *GIS4* were identified as causative genes for acetic acid tolerance through alternating ALE and UV-mutagenesis (González-Ramos et al. 2016).

Rational engineering of stress tolerance mechanisms necessitates insight into the functions of genes associated with the construction of desired phenotypes. However, the development of superior strains often requires the identification of polygenic target genes (with response to multiple CBP stress factors) to reduce the induced artificial metabolic burden via manipulation of metabolism (Brandt et al. 2021b; Mao et al. 2024). Numerous studies have contributed towards a library of gene targets with varying resistance responses to individual inhibitors. Zhang and co-workers (2019) demonstrated that the over-expression of *ADE17*, *ADE1*, and *ADE13* (involved in de novo purine biosynthesis) improved tolerance, growth, and ethanol fermentation capabilities under acetic acid and multiple inhibitor stress conditions. Moreover, the over-expression of alcohol dehydrogenases, aldehyde, and non-specific reductases was shown to confer improved growth characteristics and tolerance towards the LCB pre-treatment-derived inhibitors furfural and 5-hydroxymethylfurfural (HMF) (Cámara et al. 2022).

Evidently, the major portion of inhibitory compound-induced stress modulation is due to enzymatic detoxification, mediated by a variety of reducing and degrading proteins (Den Haan et al. 2023). However, Zhang and co-workers (2023b) recently showed that the over-expression of *HBN1*, encoding a *K. marxianus* nitroreductase, resulted in improved acetic acid, furfural, and phenol tolerance via induced reactive oxygen species (ROS) elimination. Furthermore, the deletion of *ADY2* (encoding an acetic acid transporter) resulted in improved ethanol production capabilities in the presence of acetic acid and improved growth in the presence of ethanol, acetic acid, and hydrogen peroxidase (Zhang et al. 2017).

Few studies have reported gene targets contributing to strain robustness which simultaneously improve tolerance towards secretion stress and metabolic burden. However, improved stress tolerance (acetic acid, heat, and osmotic stress) and secretion enhancement (55% increase in heterologous CBH activity) were demonstrated through over-expression of the *S. cerevisiae YHB1*, encoding a flavohemoglobin nitric oxidoreductase with known associations with oxidative stress modulation (Lamour et al. 2019). Additionally, over-expression of *ENA5* (encoding a P-type ATPase involved in the efflux of sodium) resulted in enhanced tolerance to ethanol, heat, and osmotic stress factors with a simultaneous increase in ethanol production (Wang et al. 2022). The yeast cell wall represents the first line of defense towards inhibitors and has provided multiple genes, including those encoding cell wall mannoproteins, as well as proteins involved in cell wall synthesis and remodelling, as targets for metabolic engineering aimed at enhancing tolerance towards multiple stress factors (Ribeiro et al. 2022).

Taken together, various omics-based studies have contributed to our understanding of yeast strain robustness and how it can be used to generate process-ready organisms through rational strain engineering. However, it should be noted that strain background will play a prominent role in determining the success of these interventions.

## Strain background

The choice of yeast strain background is fundamental when developing strains for CBP of lignocellulose to ethanol, as it directly impacts the strain’s ability to balance metabolic efficiency, stress tolerance, and substrate utilization (Den Haan et al. 2023). Domesticated strains, including industrial strains of *S. cerevisiae*, are well-suited for industrial ethanol production due to their robust fermentation capacity, ethanol tolerance, and ease of genetic manipulation. However, they lack the innate ability to degrade lignocellulosic substrates, necessitating significant engineering. Other potential CBP candidates include *K. marxianus*, which can grow at temperatures of 45–52 °C, produce ethanol at 38–45 °C, and utilize a broad range of sugar substrates (arabinose, galactose, mannose, xylose) commonly found in LCB (Hasunuma and Kondo 2012). Additionally, the thermotolerant methylotrophic yeast *Ogataea (Hansenula) polymorpha* can produce ethanol from glucose, cellobiose, and xylose at temperatures exceeding 37 °C. Another promising candidate is *Scheffersomyces stipitis*, a natural xylose-metabolizer, which enables it to produce ethanol from the C5 fraction of lignocellulosic biomass (Santos et al. 2016). Despite the native advantages of these alternative strains, they typically exhibit low ethanol yields and reduced tolerance to industrial stresses when compared to *S. cerevisiae* (Davison et al. 2020). Therefore, selecting or engineering a suitable yeast background often requires a balance between leveraging the advantages of wild yeasts and the industrial robustness of domesticated strains.

The choice of strain background is also critical for the phenotypic expression of any rational engineering intervention. Manipulations performed in amenable laboratory strains are less likely to translate effectively to industrial strains due to differences in their tolerance or secretion phenotypes (Den Haan et al. 2023). However, some studies have successfully conferred engineered traits to industrial strains, for example, Varize and co-workers (2022) demonstrated the over-expression of the *TRP1*, *MSN2*, and *NΔMSN2* (truncated N-terminus of *MSN2*) genes in an industrial strain (CAT-1), which were previously shown to improve ethanol tolerance in laboratory strains. When assessing fermentation capabilities, only the industrial strain over expressing *NΔMSN2* exhibited improved ethanol production. Recent studies have highlighted that natural isolates of *S. cerevisiae* may include strains better suited for second-generation (2G) ethanol production compared to those traditionally used in first-generation (1G) ethanol processes (de Witt et al. 2019a, 2019b; Jansen et al. 2018). These strains, along with others yet to be identified, could serve as superior chassis for the bioconversion of cellulose to ethanol when considering the unique demands of high protein secretion capacity, robust stress tolerance, and sustained high ethanol productivity.

## Conclusion

Despite significant progress in enabling cellulose utilization in yeast, substantial advancements are still needed to develop strains suitable for industrial CBP of LCB substrates. The inherent lack of cellulolytic enzyme production and the low protein secretion capacity of *S. cerevisiae* highlight the need for more suitable chassis strains and extensive genetic engineering to create industry-ready strains. Based on recent publications and advancements in the field, cell-tethering of cellulases appears to be a promising avenue for further exploration. Rational engineering of the cell wall itself was shown to further improve the cell-tethering of enzymes, making it an even more attractive option for CBP. Recently reported strains that produce a core set of cellulases as cell-tethered enzymes able to convert microcrystalline cellulose to ethanol also pave the way for ALE of these strains on LCB substrates to evolve strains more suited to bioconversion.

Currently, CBP-enabled yeasts still require exogenous enzymes to reach ethanol titers that are considered economically feasible. Hence, the need for further investigation into methods of improving saccharification, while still remaining economically viable, is required. The yeast secretory pathway itself contains numerous gene targets to be over-expressed or deleted to increase secretion capacity, though these must be assessed on a “trial and error” basis due to strain and protein-specific differences. Improving the protein secretory capacity can involve genetic engineering of the yeast secretory pathway from as early as transcription. Synthetic promoters show promise in mitigating the challenges associated with endogenous promoters as they provide the opportunity to tailor promoters to their specific needs and eliminate any hindering promoter elements. However, promoters must be evaluated for their suitability under process-specific conditions. Recent advancements in high throughput “omics” techniques, combined with computational biology and machine learning, have significantly enhanced our understanding of cellular processes and metabolic pathways. The development of detailed metabolic models has allowed for more accurate predictions of the impact of genetic modifications on metabolism and protein expression. High-throughput experimental workflows in biofoundries further accelerate the creation and optimization of suitable engineered strains.

The stresses encountered during industrial CBP significantly hinder the overall ethanol production capacity of yeasts. This challenge can be mitigated through rational engineering of target genes that detoxify inhibitory compounds in stressful environments or through ALE, where yeasts accumulate mutations that enhance robustness. However, strain-specific differences play a crucial role, making it essential to evaluate each strain under relevant process conditions to ensure optimal performance. Thanks to the marker-free nature of modern strain engineering tools, *S. cerevisiae* still offers significant potential for further optimization and adaptation in a CBP setting. The insights gained from these efforts will ultimately drive future advancements in the development of cellulase-producing CBP yeasts.

## Data Availability

N/A.

## Code availability

N/A.
